# Association between PFAS exposure and attention processing in adolescent boys: a pilot study

**DOI:** 10.3389/fcogn.2025.1606956

**Published:** 2025-06-13

**Authors:** Sharlene D. Newman, Yanyu Xiong

**Affiliations:** Alabama Life Research Institute, The University of Alabama, Tuscaloosa, AL, United States

**Keywords:** PFAS, EEG, attention, ADHD, brain

## Abstract

Perfluorinated alkylated substances (PFAS) are a group of chemicals that have been used in industries and products for decades. Little is known about the neurological effects of PFAS in humans, particularly during adolescence, which is a critical period for brain development, making exposure to environmental toxins during this period even more likely to lead to cognitive deficits. We recruited adolescent boys to participate in this pilot study. We performed community data collection of (1) blood sample to measure blood-level PFAS, (2) parental assessments of behavior, and (3) electroencephalography (EEG) data during the performance of an attention task. Our findings demonstrated the feasibility of collecting community data, including EEG data. The data collected revealed an association between PFAS levels and EEG measures of attention, specifically P2 and N2, and parental assessment of attention. Although this is a pilot study, it indicates the feasibility of conducting more comprehensive studies to examine the neurocognitive effects of PFAS exposure.

## Introduction

Perfluorinated alkylated substances (PFAS) are a group of chemicals that have been used in industries and consumer products since the 1950s. Studies show that between 97% and 99% of the US population have detectable levels of PFAS in their blood (ATSDR, 2021; Lewis et al., 2015). PFAS are often called “forever chemicals” due to their resistance to degradation and their ability to bioaccumulate in human tissue, with half-lives ranging from 4 to 6 years (Olsen et al., 2007). These substances have been linked to a range of adverse health effects, including reduced immune responses, cancer, and pregnancy-related complications (National Academies of Sciences, 2022a). The Environmental Protection Agency (EPA), Centers for Disease Control and Prevention (CDC), and National Center for Environmental Health (NCEH) consider human exposure to PFAS a significant national health concern.

While the adverse health outcomes of PFAS exposure are well-documented, their neurological effects, particularly in adolescents, remain underexplored. Some studies have reported associations between PFAS exposure and cognitive outcomes (Harris et al., 2021). For instance, cross-sectional research has linked PFAS exposure to behavioral and executive functioning problems in children (Gump et al., 2011; Hoffman et al., 2010; Vuong et al., 2018). National Health and Nutrition Examination Survey (NHANES) cohort studies (1999–2000 and 2003–2004) identified an increased prevalence of ADHD in adolescents exposed to PFAS (Lewis et al., 2015). Additional findings suggest that children who are exposed to PFAS may exhibit heightened impulsivity (Harris et al., 2021) and both internalizing and externalizing behavioral issues (Girardi et al., 2023).

This pilot study focuses on the feasibility of examining the neurocognitive effects of PFAS exposure on attentional processing in adolescents. Previous studies have shown that PFAS can cross the blood–brain barrier and accumulate (Wang et al., 2018; Greaves et al., 2013) in brain regions such as the hippocampus (Cao and Ng, 2021). Long et al. (2013) reported increased hippocampal glutamate levels—linked to ADHD (Elia et al., 2020; Ugarte et al., 2023)—following PFAS exposure. In our study, we collected blood samples to measure PFAS levels, electroencephalography (EEG) data during an attention task (Flanker task), and parental assessments of attention.

## Methods

### Participants

A total of 18 adolescent boys (mean age = 11.7 ± 1.8 years; all African American) participated in the study. Parental consent and adolescent assent were obtained with the approval of the Institutional Review Board of the University of Alabama.

### Procedure

Data were collected in the participants' communities, with 16 participants from McIntosh, Alabama, and two from Tuscaloosa, Alabama. Blood samples were collected from each participant using child-friendly Eurofins, Sacramento, CA, USA (https://www.eurofinsus.com/environment-testing/pfas-testing/services/blood-and-serum/) self-collection kits (finger-prick method). The samples were sent to Eurofins for PFAS analysis. The analysis included the following CDC NHANES analytes: PFOA, PFOS, PFHxS, PFUnA, and a total PFAS level. The PFOS levels were used in the analysis.

The SNAP-IV 26 (Swanson et al., 2001, 1999), an assessment battery designed to assess attention in children aged 8–18 years, was completed by guardians. The test items are based on the *DSM-IV* criteria for inattention, hyperactivity/impulsivity, and oppositional defiant disorder (ODD).

**Figure 1:**
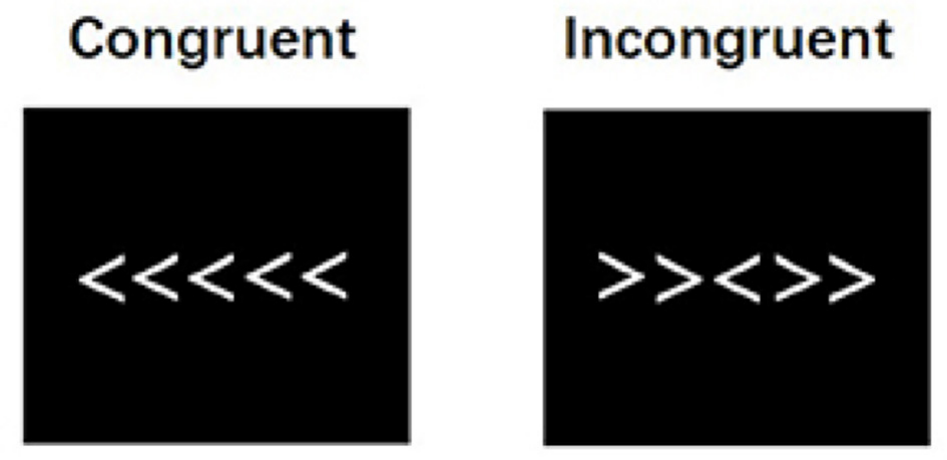
Sample trials of the Flanker task.

Participants also completed the Erikson Flanker task during the EEG data collection. The Flanker task assesses selective attention and inhibitory function. The task included one target arrow in the middle and two flanker arrows on each side pointing in the same (congruent) or opposite (incongruent) direction with the target arrow (Eriksen and Eriksen, 1974; see Figure 1). Each trial began with a fixation (1,000 ms) and was presented on a Lenovo laptop (IntelCore i5) installed with Psychopy (version 2023.2.3). A row of five arrows was presented with a start jitter ranging from 95 to 105 ms to avoid eliciting neural oscillations entrained with the trial presentation frequency. Participants indicated whether the middle target arrow points to the left or right by pressing the left or right arrow key on the keyboard. Visual feedback was provided after each trial. A total trial of 504 was split into two blocks, with 126 congruent and 126 incongruent trials randomly presented in each block. EEG experimental sessions were ~30 min.

### EEG data collection

EEG data were recorded using a 128-channel Geodesic Sensor Net (Tucker, 1993), a Power Macintosh iMac computer (3.2 GHz Quad-Core Intel Xeon) installed with Net Station (version 4.5.6; Acquisition, Viewer, and Waveform Tools), and an EGI NetAmps400 amplifier (EGI, Eugene, OR) (https://www.magstim.com/magstimegi-eeg/). The impedance of all electrodes was <50 kΩ at the beginning of the recording. The signals were sampled at 1,000 Hz and filtered from DC to 250 Hz offline. Data collection occurred in a local church for 10 participants and at the University of Alabama for two participants. We have experience using the mobile EEG system with African American participants (Hudac et al., 2022).

### Data preprocessing

MNE-python (version 1.6.0, python 3.9) (Gramfort et al., 2013) was used to import the raw mff EEG data of each subject and notch filter the 60 Hz line noise before removing the bad segments with muscle artifacts and the channels consistently bad over 10% of the raw data set. The independent component analysis (FastICA) with maximum iterations of 1,000 was employed to remove components related to eye movements and cardiac artifacts based on the comprehensive evaluation of the typographic maps, power spectral density plots, and trial-wise plots. Each data set was then referenced to the grand average after channel interpolation. The continuous EEG time series was divided into epochs of 550 ms (−200 to 350 ms). The baseline correction was performed using the 200 ms before each trial. Epochs with an amplitude above 100e-6 μV and a post-stimulus duration <100 ms were removed from both the behavioral and EEG data sets as previous studies have reported that the major neural signatures related to perception and attention occur between 50 and 300 ms after the stimulus onset (Woodman, 2010). The total number of congruent and incongruent trials is 252, respectively. The average rejection rate is 15.1% across conditions. There is no significant difference in the rejection rate between conditions (congruent: mean = 38.7; incongruent: mean = 37.2; *t* = 0.76, *p* > 0.05). The average number of clean trials in the congruent condition was 218 across participants and 220 in the incongruent condition. There is no significant difference in the clean trial numbers between the two conditions (*t* = 2.09, *p* > 0.05). The epochs of (in)congruent conditions for each participant were averaged across all electrodes.

### ERP analysis

**Figure 2:**
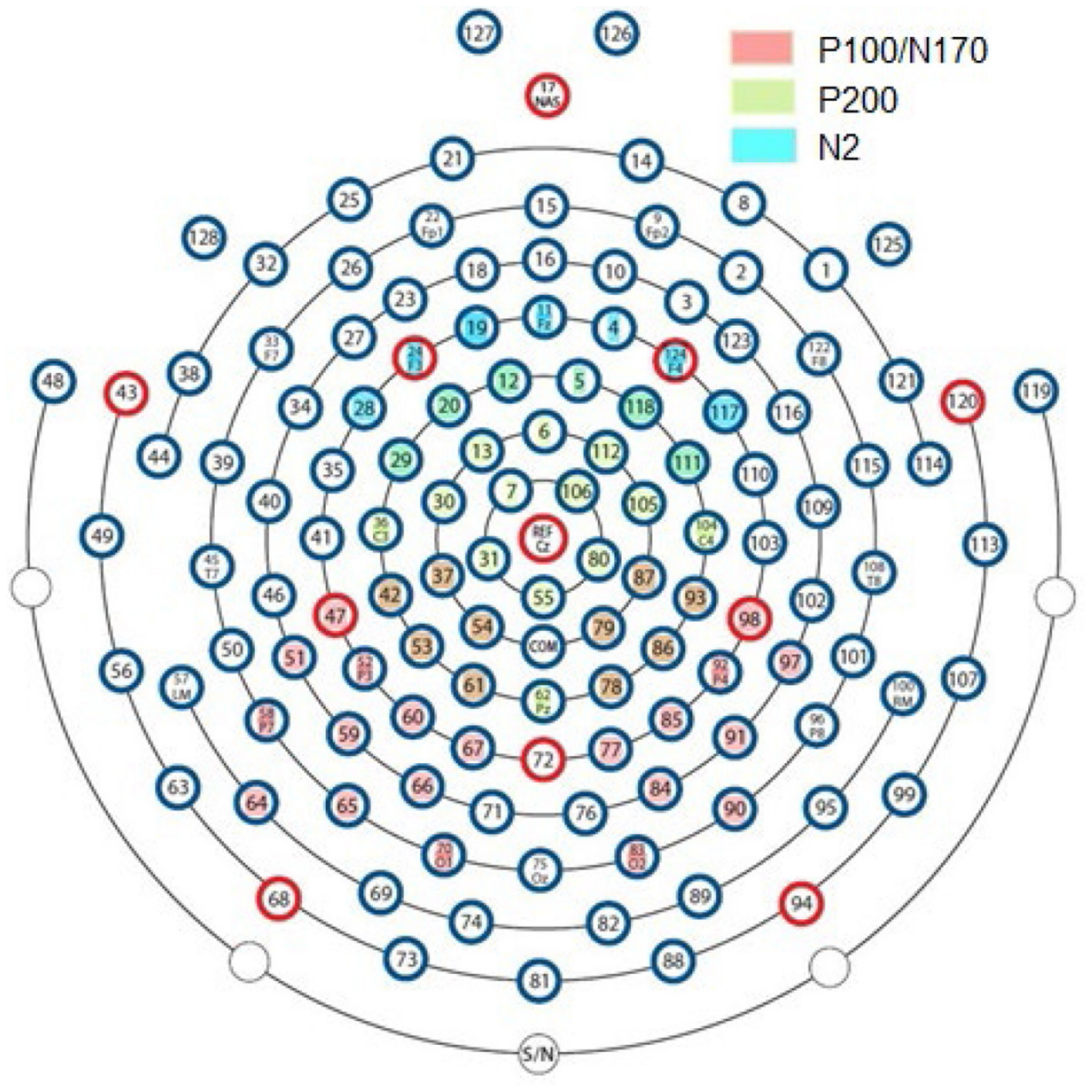
Electrodes included in each region of interest.

Three windows were selected to examine the stimulus-locked ERPs and calculate the averages of each condition for the bilateral P1 (50–180 ms), N170 (130–250 ms), and the central P2 (100–250 ms), and bilateral frontal N2 (300–500 ms) components (Hileman et al., 2011; Negrini et al., 2017) for each ERP component on the selected electrodes in the region of interest (ROI; Figure 2). The P1 and N170 components include two posterior ROIs with 15 electrodes bilaterally (Figure 2). P2 is mapped by a single central ROI around Cz with 29 electrodes. N2 includes 13 frontal electrodes as ROIs.

## Results

Two participants without blood PFAS data and one participant with a level more than two standard deviations above the mean were excluded from the analysis, resulting in a final group of 15 participants with PFAS data. The blood PFAS levels ranged from 0 to 3.27 ng/ml (*M* = 1.77 ± 0.69). It is important to note that there is potential for adverse effects in sensitive populations, such as children, at levels between 2 and 20 ng/ml (National Academies of Sciences, 2022b).

**Figure 3:**
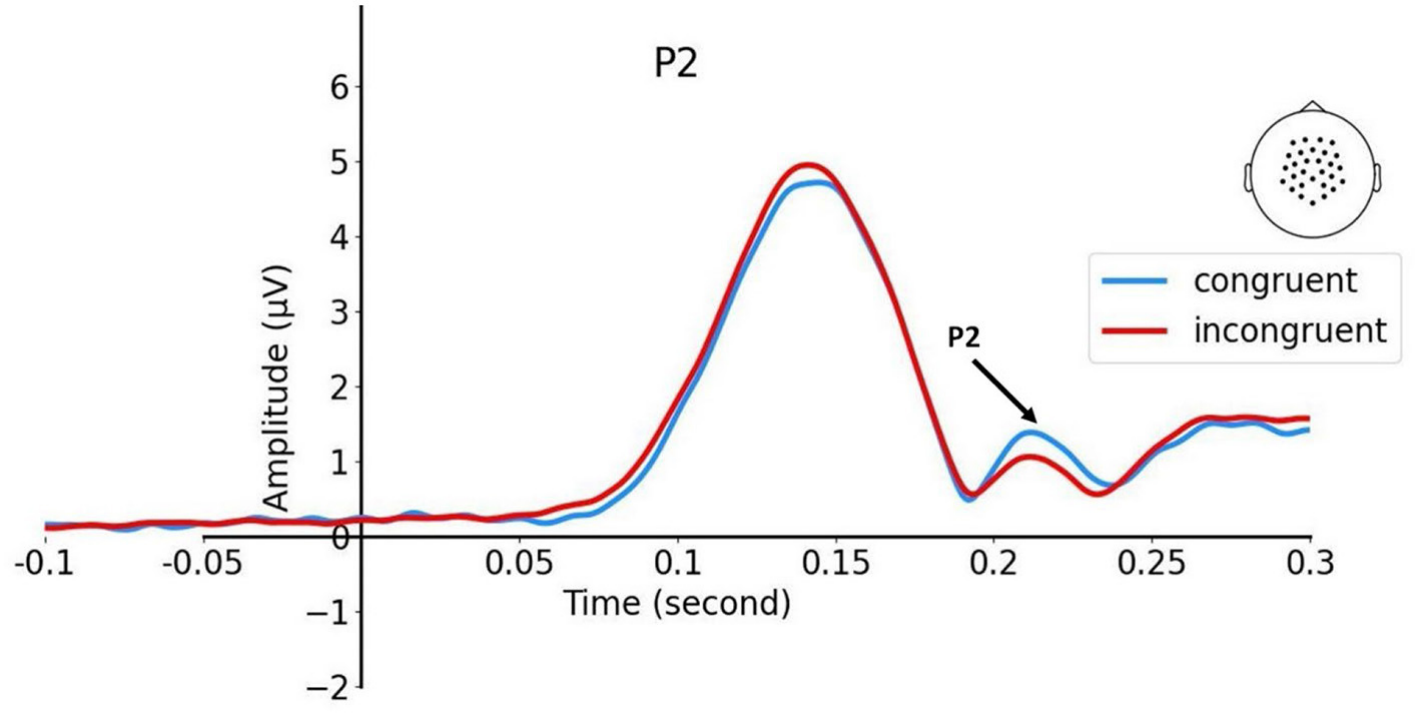
Depicts the ERP time course showing the effect of congruency at P2. The electrode cluster is also shown.

When examining the effect of congruency, EEG data (*N* = 18; Figure 3) showed a significant P2 response modulation [two-tailed *t*_(17)_ = 3.49, *p* = 0.003], reaction time [congruent: *M* = 0.95 s; incongruent: *M* = 1.36 s; two-tailed *t*_(17)_ = 9.84, *p* < 0.0001], and accuracy [congruent: *M* = 94.6%; incongruent: *M* = 84.8%; two-tailed *t*_(17)_ = 2.99, *p* = 0.008]. The whole-head topographic map of the conditional differences revealed that the ERP amplitude of the congruent trials was more positive than incongruent trials in the centro-parietal region.

A regression analysis was performed using the ERP incongruent-congruent difference as the outcome variable and PFOS levels, total SNAP-IV score, and their interaction as predictors. The results indicated that the N2 component (both left and right) and P2 component showed effects related to PFOS levels, attention as measured by SNAP-IV scores, and the interaction between these two factors. In addition, a regression analysis was performed with only PFOS as the predictor variable. The left and right N2 responses showed significant effects of PFOS (*p* =*0.0*43 and 0.044, respectively); however, it was not significant for the P2 response (*p* = 0.42; Table 1).

**Table 1:** Regression results.

**Variable**	**P2**	**N2 (left)**	**N2 (right)**
*F, p*-value	*F*_(3, 11)_ = 3.78, *p* = 0.044	*F*_(3, 11)_ = 9.12, *p* = 0.0025	*F*_(3, 11)_ = 12.48, *p* = 0.0007
*R* ^2^	0.51	0.71	0.77
PFOS	β = 1.01E-6, *t* = 2.48, *p =* 0.031	β = 2.15E-6, *t* = 2.57, *p =* 0.0262	β = 1.75E-6, *t* = 3.14, *p =* 0.0093
SNAP-IV	β = 9.43E-8, *t* = 2.88, *p =*0.015	β = 2.5E-7, *t* = 3.85, *p =* 0.0027	β = 2.03E-7, *t* = 4.55, *p =* 0.0008
Interaction	β = −7.95E-8, *t* = −3.09, *p =* 0.01	β = −2.13E-7, *t* = −4.03, *p =* 0.002	β = −1.7E-7, *t* = −4.81, *p =* 0.0005

## Discussion

This pilot study establishes the feasibility of investigating the neurocognitive effects of PFAS exposure using mobile EEG and minimally invasive blood sampling in rural communities. Despite the limited sample size and the exploratory nature of the study, our findings suggest potential associations between PFAS exposure and attention-related brain activity.

Specifically, the N2 component, linked to stimulus–response conflict (Veen and Carter, 2002), and the P2 component, associated with selective attention (Kałamała et al., 2018), showed relationships with PFAS levels and SNAP-IV scores. The observed interaction effects further emphasize the need for larger studies.

Unlike EEG and behavioral data, SNAP-IV is based on parental reporting and is inherently more subjective. Nevertheless, the interaction between PFAS levels and SNAP-IV results underscores its potential value in complementing objective data. Our findings align with previous research, including the meta-analysis by Yao et al. (2023) linking PFAS to ADHD and the study by Girardi et al. (2022) associating PFAS in tap water with increased aggressive behavior.

One limitation of the study is that the sample is limited in size and diversity, as it consisted entirely of African American boys. We did not obtain socioeconomic and environmental measures that may affect brain functioning. Future studies should include a more diverse sample, specifically female participants, and more comprehensive measures of participants' environments and cognitive abilities.

Future research should expand on these findings by including larger and more diverse samples, exploring longitudinal designs, and clarifying the neural mechanisms through which PFAS affects cognition. The success of community-based data collection in this study highlights the potential of including underserved populations in neurocognitive research.

## Data Availability

The raw data supporting the conclusions of this article will be made available by the authors, without undue reservation.

## References

[B1] ATSDR (2021). Toxilogical Profile for Perfluoroalkys. ATSDR, Division of Toxicology and Human Health Sciences, Centers for Disease Control. Available online at: https://www.atsdr.cdc.gov/toxprofiles/tp200.pdf (accessed September 22, 2024).

[B2] Cao Y. Ng C. (2021). Absorption, distribution, and toxicity of per-and polyfluoroalkyl substances (PFAS) in the brain: a review. Environ. Sci. Process. Impacts. 23, 1623–1640. 10.1039/D1EM00228G 34533150

[B3] Elia J. Izaki Y. Ambrosini A. Hakonarson H. (2020). Glutamatergic neurotransmission in ADHD: neurodevelopment and pharmacological implications. J. Pediatr. Neonatol. 2:1006. 10.1038/tp.2014.11 24643164 PMC3966039

[B4] Eriksen B. A. Eriksen C. W. (1974). Effects of noise letters upon the identification of a target letter in a nonsearch task. Percept. Psychophys. 16, 143–149. 10.3758/BF03203267

[B5] Girardi P. Lupo A. Mastromatteo L. Y. Scrimin S. (2022). Mothers living with contamination of perfluoroalkyl substances: an assessment of the perceived health risk and self-reported diseases. Environ. Sci. Pollut. Res. 29, 60491–60507. 10.1007/s11356-022-20085-5 35426015

[B6] Girardi P. Lupo A. Mastromatteo L. Y. Scrimin S. (2023). Behavioral outcomes and exposure to perfluoroalkyl substances among children aged 6–13 years: the TEDDY child study. Environ. Res. 231:116049. 10.1016/j.envres.2023.116049 37207732

[B7] Gramfort A. Luessi M. Larson E. Engemann D. A. Strohmeier D. Brodbeck C.. (2013). MEG and EEG data analysis with MNE-Python. Front. Neurosci. 7:267. 10.3389/fnins.2013.00267 24431986 PMC3872725

[B8] Greaves A. K. Letcher R. J. Sonne C. Dietz R. (2013). Brain region distribution and patterns of bioaccumulative perfluoroalkyl carboxylates and sulfonates in East Greenland polar bears (*Ursus maritimus*), Environ. Toxicol. Chem. 32, 713–722. 10.1002/etc.2107 23280712

[B9] Gump B. B. Wu Q. Dumas A. K. Kannan K. (2011). Perfluorochemical (PFC) exposure in children: associations with impaired response inhibition. Environ. Sci. Technol. 45, 8151–8159. 10.1021/es103712g 21682250 PMC3184198

[B10] Harris M. H. Oken E. Rifas-Shiman S. L. Calafat A. M. Bellinger D. C. Webster T. F.. (2021). Prenatal and childhood exposure to per-and polyfluoroalkyl substances (PFAS) and child executive function and behavioral problems. Environ. Res. 202:111621. 10.1016/j.envres.2021.111621 34237332 PMC11318511

[B11] Hileman C. M. Henderson H. Mundy P. Newell L. Jaime M. (2011). Developmental and individual differences on the P1 and N170 ERP components in children with and without autism. Dev. Neuropsychol. 36, 214–236. 10.1080/87565641.2010.549870 21347922 PMC3724226

[B12] Hoffman K. Webster T. F. Weisskopf M. G. Weinberg J. Vieira V. M. (2010). Exposure to polyfluoroalkyl chemicals and attention deficit/hyperactivity disorder in US children 12–15 years of age. Environ. Health Perspect. 118, 1762–1767. 10.1289/ehp.1001898 20551004 PMC3002197

[B13] Hudac C. M. Wallace J. S. Ward V. R. Friedman N. R. Delfin D. Newman S. D.. (2022). Dynamic cognitive inhibition in the context of frustration: increasing racial representation of adolescent athletes using mobile community-engaged EEG methods. Front. Neurol. 13:918075. 10.3389/fneur.2022.918075 36619932 PMC9812645

[B14] Kałamała P. Szewczyk J. Senderecka M. Wodniecka Z. (2018). Flanker task with equiprobable congruent and incongruent conditions does not elicit the conflict N2. Psychophysiology 55:e12980. 10.1111/psyp.12980 28845513

[B15] Lewis R. C. Johns L. E. Meeker J. D. (2015). Serum biomarkers of exposure to perfluoroalkyl substances in relation to serum testosterone and measures of thyroid function among adults and adolescents from NHANES 2011-2012. Int. J. Environ. Res. Public Health 12, 6098–6114. 10.3390/ijerph120606098 26035660 PMC4483690

[B16] Long Y. Wang Y. Ji G. Yan L. Hu F. Gu A.. (2013). Neurotoxicity of perfluorooctane sulfonate to hippocampal cells in adult mice. PLoS ONE 8:e54176. 10.1371/journal.pone.0054176 23382877 PMC3559704

[B17] National Academies of Sciences Engineering, and Medicine. (2022a). Guidance on PFAS *Exposure, Testing and Clinical Follow-Up*. Washington, DC: The National Academies Press. 35939564

[B18] National Academies of Sciences Engineering, and Medicine.. (2022b). “PFAS testing and concentrations to inform clinical care of exposed patients,” in Guidance on PFAS Exposure, Testing, and Clinical Follow-Up. Washington, DC: National Academies Press (US).

[B19] Negrini M. Brkić D. Pizzamiglio S. Premoli I. Rivolta D. (2017). Neurophysiological correlates of featural and spacing processing for face and non-face stimuli. Front. Psychol. 8:333. 10.3389/fpsyg.2017.00333 28348535 PMC5346548

[B20] Olsen G. W. Burris J. M. Ehresman D. J. Froehlich J. W. Seacat A. M. Butenhoff J. L.. (2007). Half-life of serum elimination of perfluorooctanesulfonate, perfluorohexanesulfonate, and perfluorooctanoate in retired fluorochemical production workers. Environ. Health Perspect. 115, 1298–1305. 10.1289/ehp.10009 17805419 PMC1964923

[B21] Swanson J. Lerner M. March J. Gresham F. M. (1999). Assessment and intervention for attention-deficit/hyperactivity disorder in the schools: lessons from the MTA study. Pediatr. Clin. North Am. 46, 993–1009. 10.1016/S0031-3955(05)70168-1 10570701

[B22] Swanson J. M. Kraemer H. C. Hinshaw S. P. Arnold L. E. Conners C. K. Abikoff H. B.. (2001). Clinical relevance of the primary findings of the MTA: success rates based on severity of ADHD and ODD symptoms at the end of treatment. J. Am. Acad. Child Adolesc. Psychiatry 40, 168–179. 10.1097/00004583-200102000-00011 11211365

[B23] Tucker D. M. (1993). Spatial sampling of head electrical fields: the geodesic sensor net. Electroencephalogr. Clin. Neurophysiol. 87, 154–163. 10.1016/0013-4694(93)90121-B 7691542

[B24] Ugarte G. Piña R. Contreras D. Godoy F. Rubio D. Rozas C.. (2023). Attention deficit-hyperactivity disorder (ADHD): from abnormal behavior to impairment in synaptic plasticity. Biology 12:1241. 10.3390/biology12091241 37759640 PMC10525904

[B25] Veen V. V. Carter C. S. (2002). The timing of action-monitoring processes in the anterior cingulate cortex. J. Cogn. Neurosci. 14, 593–602. 10.1162/08989290260045837 12126500

[B26] Vuong A. M. Yolton K. Wang Z. Xie C. Webster G. M. Ye X.. (2018). Childhood perfluoroalkyl substance exposure and executive function in children at 8 years. Environ. Int. 119, 212–219. 10.1016/j.envint.2018.06.028 29980044 PMC7442288

[B27] Wang J. Pan Y. Cui Q. Yao B. Wang J. Dai J. (2018). Penetration of PFASs across the blood cerebrospinal fluid barrier and its determinants in humans. Environ. Sci. Technol. (52, 13553–13561. 10.1021/acs.est.8b04550 30362723

[B28] Woodman G. F. (2010). A brief introduction to the use of event-related potentials in studies of perception and attention. Atten. Percept. Psychophys. 72, 2031–2046. 10.3758/BF03196680 21097848 PMC3816929

[B29] Yao H. Fu Y. Weng X. Zeng Z. Tan Y. Wu X.. (2023). The association between prenatal per-and polyfluoroalkyl substances exposure and neurobehavioral problems in offspring: a meta-analysis. Int. J. Environ. Res. Public Health 20:1668. 10.3390/ijerph20031668 36767045 PMC9914055

